# Results of 167 consecutive cases of acetabular fractures using the Kocher-Langenbeck approach: a case series

**DOI:** 10.1186/s13018-017-0563-6

**Published:** 2017-04-26

**Authors:** Lukas L. Negrin, David Seligson

**Affiliations:** 1 Department of Trauma Surgery, Medical University of Vienna, Waehringer Guertel 18 – 20, 1090 Vienna, Austria; 2 Fracture Service of the Department of Orthopedic Surgery, University of Louisville Hospital, 530 S. Jackson Street, Louisville, KY 40202 USA

**Keywords:** Acetabular fractures, Kocher-Langenbeck, Posterior approach, Outcome

## Abstract

**Background:**

Acetabular fractures are quite challenging injuries for the orthopedic surgeon because of their low incidence and their deep and complex anatomy. The objective of this study was to evaluate surgeon-independent parameters that might influence radiographic outcome and early complication rates of high-energy acetabular fractures treated by open reduction and internal fixation via the Kocher-Langenbeck approach, the golden standard for posterior access.

**Methods:**

One hundred sixty-seven consecutive patients (111 males and 56 females) with a mean age of 41.8 years and a mean follow-up period of 10 months were surgically treated by one experienced surgeon at a level I trauma center within 10 years. To quantify the radiographic outcome, the Matta, Brooker, and Epstein grades were used. Posttraumatic arthritis and avascular necrosis of the femoral head (defined as Helfet grades 3 or 4 and Ficat/Arlet stages 3 or 4, respectively) were evaluated. Furthermore, subgroup analyses according to fracture type, age, and gender were performed for each outcome measure and complication (infection, hemorrhagic shock, revision surgery, nerve damage, and need of a total hip arthroplasty).

**Results:**

65 A1, 34 A2, 51 B1, and 17 B2 fractures were identified according to the AO/ASIF classification. Of all patients, reduction was rated anatomic in 63.5%, imperfect in 22.2%, and poor in 14.4%. Degenerative changes were observed in 49.7%; 37.9% were affected by heterotopic ossification, 21.6% by posttraumatic arthritis, and 5.4% by avascular necrosis of the femoral head. Fifteen percent were diagnosed with a nerve damage, and 4.8% sustained an infection. Total hip arthroplasty was performed in 10.2%. Revision surgery due to secondary loss of reduction, seroma/hematoma, and wound infection was indicated in 6.0%.

**Conclusions:**

Fracture type, age, and gender are prognostic factors for the surgical outcome after ORIF of high-energy acetabular fractures.

**Electronic supplementary material:**

The online version of this article (doi:10.1186/s13018-017-0563-6) contains supplementary material, which is available to authorized users.

## Background

Acetabular fractures are rare injuries. Three out of 100,000 inhabitants are estimated to suffer one each year [1]. Due to the fact that the average age of the population is growing and that the elderly patients are maintaining an active lifestyle, the incidence of acetabular fractures in the elderly is expected to rise [2]. Most of the acetabular fractures result from high-energy trauma like motor vehicle accidents (80.5%) or falls from a significant height (10.7%) [3]. They are generally considered as serious injuries and often occur in multiple trauma patients [3]. In contrast, fragility acetabular fractures in osteoporotic patients can be caused by simple low-energy falls from standing height [4]. Type and displacement of an acetabular fracture are determined by the position of the femur at the time of impact and the direction of the force. For instance, dashboard injuries cause posterior wall fractures of the acetabulum because the force is applied to the flexed knee and hip. Alternatively, in case of a collision between a motor vehicle and a pedestrian, the force can be oriented directly to the greater trochanter, resulting in transverse and anterior types of acetabular fractures [5]. Over the past 40 years, the management of displaced acetabular fractures has changed from conservative to operative [3]. Nowadays, open reduction and internal fixation (ORIF) is the standard therapy for acetabular fractures in case of a displacement of more than 2 mm [6]. In general, a satisfactory radiographic and clinical outcome can be expected after anatomic reduction [7]. Clinical outcome has been reported to decrease with increasing patient age [8, 9], while other researchers could not confirm these findings [10–12]. Fracture pattern is often [8, 13, 14] but not necessarily [11] considered as a significant predictor of radiographic and functional results. However, gender is not supposed to influence the outcome after acetabular surgery [11, 12, 15], whereas early ORIF can be assumed to result in a higher likelihood of excellent or good radiographic results [8, 13, 16].

Infections in patients treated with ORIF may be devastating because they may lead to additional surgery, poorer healing rates, and decreased functional and psychological outcomes. Endogenous infections are caused by microorganisms originated from the patient’s skin or a perforated viscus, whereas exogenous infections occur when microorganisms from the environment contaminate a traumatic wound. The cause of infection may be related either to the initial injury or to the soft tissue damage, and the lymphatic and osseous trauma provoked by ORIF. Of all hospital-acquired infections, surgical site infections are the most common and cost-intensive ones [17], occurring despite of antibiotic prophylaxis and sterile surgical techniques. They are caused by bacteria that invade through the incisions during surgery and may develop near the skin surface around an incision or deep in the surgical wound, thus representing serious complications [18]. Risk factors for surgical site infections include extended preoperative hospitalization, massive intraoperative blood loss, larger amount of packed red blood cell unit transfused, and prolonged surgery time [18, 19].

In general, fractures due to high-energy trauma may provoke extensive external and/or internal bleeding resulting in a hemorrhagic shock, characterized by decreased cardiac output and inadequate tissue perfusion, which can lead to life-threatening conditions such as metabolic acidosis or irreversible brain, hepatic, and renal damage [20]. Because of the extensive blood supply of the pelvic bone, acetabular fractures may be associated with bleeding from the bone itself and/or a retroperitoneal hematoma that may cause severe hemorrhagic shock several hours after the injury [21, 22].

Over time, various standard and extensile approaches have been established for the treatment of a given acetabular fracture. Although the Kocher-Langenbeck approach [23] is considered as the golden standard for posterior access [3], studies dealing solely with this approach are rare [24–27], including a relatively small number of patients that ranges from 27 to 104. Undoubtedly, surgeon’s expertise is a crucial factor [28]. Due to the anatomic location and the three-dimensional structure of the bone, acetabular fracture surgery is extremely challenging for orthopedic surgeons. The practical skills of a surgeon play an important role in achieving favorable treatment outcomes. Furthermore, his/her experience is the decisive factor for the choice of the most appropriate approach [29] and the optimal timing of surgery [30] that has to comply with patient’s comorbidities and concomitant injuries.

Due to the fact that both surgeon’s expertise and the selected approach to a given acetabular fracture may influence the outcome, the objective of our retrospective case series was to identify surgeon-independent and approach-specific parameters as prognostic factors for radiographic outcome and early complication rates of acetabular fractures treated via the Kocher-Langenbeck approach.

## Methods

Our retrospective study focused on the radiographic outcome and early complications of all trauma victims (1) suffering high-energy fractures, (2) who had been treated with ORIF (3) by a single surgeon, the senior author D.S., (4) at a single level I trauma center (5) within a time period of 10 years (6) through an isolated Kocher-Langenbeck approach. Exclusion criteria included manifestation of severe osteoporosis, low-energy trauma, pathological fractures, and previous history of hip injuries, as well as dementia and other disease processes, which made postoperative compliance unreliable. Additionally, those patients were excluded, who were lost from the outpatient follow-up, and those, whose medical files and/or imaging examinations presented deficiencies among the data taken into account in this study. D.S. is an experienced long-serving trauma surgeon; he had completed the learning curve already well before the surgeries of the patients evaluated in this study were performed. According to fracture type, location of maximal displacement and soft tissue conditions, he considered either the prone or the lateral position appropriate for ORIF. The AO/ASIF classification [31] of the fractures and the radiographic result of reconstruction were ascertained from standard radiographs of the hip (a.p., ala, obturator) and from CT scans. To quantify the radiographic outcome, several criteria were used. According to Matta [8], the quality of fracture reduction was recorded in millimeters and graded as the maximum residual displacement of the fracture of any of the three standard radiographs of the hip. Anatomic reduction was defined as up to 1 mm displacement. If the displacement was in the range of 2 to 3 mm, it was categorized as imperfect reduction, and if it was more than 3 mm, it was categorized as poor reduction. The Epstein grades [32] assessed increased wear due to fracture and reduction malalignment, rating femoral head-acetabular relationship with regard to degenerative changes. The degree of heterotopic ossification was documented by the Brooker [33] et al. classification scheme. Finally, Helfet [34] grades 3 or 4 and Ficat/Arlet [35] stages 3 or 4 defined posttraumatic arthritis and avascular necrosis of the femoral head (AVN), respectively. Data collected by medical records included patient gender and age, side of injury, time from accident to surgery, time needed to position the patient, length of surgery, estimated intraoperative blood loss, need for revision or secondary surgery, follow-up period, and documented complications. Postoperative hypovolemic shock was diagnosed by a shock index (the ratio of heart rate to systolic blood pressure [36]) higher than 1 [37].

Statistical analysis was mainly conducted using IBM SPSS Statistics Version 23, 64 bit. The Kolmogorow-Smirnow test was performed to assess normal distribution of the samples. For normally distributed parameters, mean and standard deviation are displayed. For skew distribution, parameters are presented as median and interquartile range (IQR). Student’s *t* tests were applied to compare normally distributed metric parameters, whereas continuous variables of a skew distribution were matched using the Mann-Whitney-Wilcoxon rank-sum test for unrelated samples. Group-differences in nominal data between several categories were analyzed by means of cross tabulation and Pearson’s **c**hi-square test. In general, *p* values lower than 0.05 were considered to be statistically significant. Statistical power 1-β was calculated by means of G* Power Version 3.1. [38].

## Results

A total of 167 consecutive patients underwent surgical treatment; 68 procedures were performed in the lateral position and 99 in the prone position. Our patient population included 111 (66.5%) men and 56 (33.5%) women with a mean age of 41.8±15.1 (range, 14 to 85) years and a median follow-up period of 7 (IQR = 5–13; range, 3 to 84) months. The side of injury was left in 75 patients and right in 92 patients. The median time from accident to surgery was 4 (IQR = 3–7, range, 0 to 25) days. The mean time before incision, defined for this study as the time from patient entering the operating room to skin incision, was 60±20 (range, 15 to 135) min. The surgeries lasted 157±45 (range, 75 to 290) min on average, and the median estimated intraoperative blood loss was 300 (IQR = 200–600; range, 30 to 2000) mL. Sixty-five (38.9%) A1 fractures, 34 A2 (20.4%), 51 B1 (30.5%), and 17 B2 (10.2%) fractures were identified. Anatomic reduction was achieved in 106 patients (63.5%), imperfect and poor reduction was achieved in 37 (22.2%) and 24 (14.4%) patients, respectively.

**Fig. 1:**
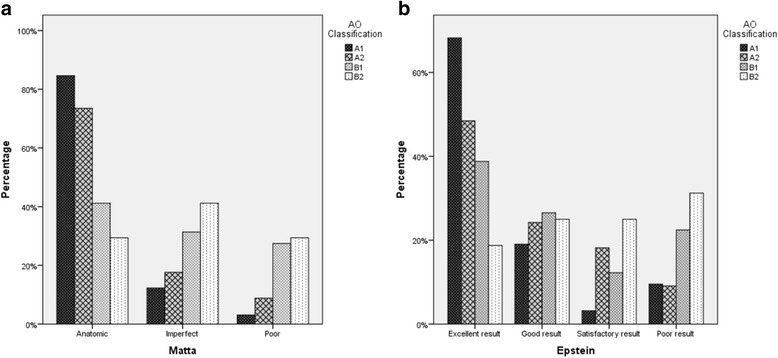
**a** Reduction according to Matta and **b** degenerative changes according to Epstein

Figure 1a displays the quality of reduction depending on the fracture type. The more difficult the fracture the worse the reduction was rated according to Matta (*p* = 0.000004; 1-β = 0.99997). A similar distribution is shown for the Epstein grades in Fig. 1b (*p* = 0.005; 1-β = 0.998). Degenerative changes were observed in 49.7% of the patients (grade I changes, good result 23.0%, grade II changes, satisfactory result 11.2%, grad III changes, poor result 15.5%). Of the heterotopic ossification, no significant impact of the fracture type was revealed (*p* = 0.095). In total, 37.9% of the patients were affected (grad I 26.1%, grade II 6.2%, and grade III 5.6%).

Posttraumatic arthritis was found in 36 patients (21.6%). Concerning AVN, nine patients (5.4%) were affected. In all cases, AVN was caused by posterior dislocation of the hip during the accident without any Pipkin fracture. Fifteen patients (9.0%) were diagnosed with a temporary nerve damage (traumatic 9, iatrogenic 6) and 10 patients (6.0%) with a persistent nerve damage (traumatic 7, iatrogenic 3). Eight patients (4.8%) sustained an infection. Four had methicillin-sensitive Staphylococcus aureus (MSSA) infections of the blood (sepsis), two had a methicillin-resistant Staphylococcus aureus (MRSA) infection of the bone (osteomyelitis), and two had an MRSA osteomyelitis together with a Pseudomonas infection of the wound (cellulitis). Postoperative hemorrhagic shock was detected in 26 patients (15.6%). Revision surgery due to secondary loss of reduction was indicated in five patients. Their demographic data are presented in Table 1.

**Table 1:** Demographic data of patients with secondary loss of reduction

Sex	AO classification	Interval accident surgery (days)	Length of surgery (min)	Matta grades	AVN
Male	B2	4	150	Imp.	Yes
Male	B2	3	260	Poor	Yes
Male	A1	3	164	Poor	No
Female	B1	6	187	Imp	Yes
Male	A2	10	159	Anat.	No

Furthermore, revision surgery was necessary in one patient due to seroma/hematoma and in four patients due to wound infection. Total hip arthroplasty (THA) was performed in 17 patients (10.2%), 11.2 (range, 0.5 to 60) months after initial surgery. In one patient, osteomyelitis developed after ORIF of an A2 fracture; the plate had to be removed 5 years postoperatively. Concerning the fracture types, there were no significant differences due to infection, nerve damage, avascular necrosis of the femoral head as well as revision surgery and THA needed (*p* ≥ 0.13). Solely, the incidence of posttraumatic arthritis and the occurrence of postoperative hemorrhagic shock depended on the fracture type (*p* = 0.004; *p* = 0.040). The respective numbers of patients and the relevant percentages are presented in Table 2. No deep venous thrombosis, pulmonary embolism, malposition of the implant, nonunion, or implant dysfunction was noted.

**Table 2:** Number of complications referring to fracture type

	A1	A2	B1	B2
Number of patients	65	34	51	17
Infection	4 (6.2%)	3 (8.8%)	1 (2.0%)	0 (0%)
Nerve damage	7 (10.8%)	4 (11.8%)	9 (17.6%)	5 (29.4%)
Revision surgery	4 (6.2%)	3 (8.8%)	1 (2.0%)	2 (11.8%)
THA	4 (6.2%)	2 (5.9%)	9 (17.6%)	2 (11.8%)
AVN	1 (1.5%)	1 (2.9%)	5 (9.8%)	2 (11.8%)
*Posttraumatic arthritis*	*6 (9.2*%*)*	*7 (20.6*%*)*	*16 (31.4*%*)*	*7 (41.2*%*)*
*Hemorrhagic shock*	*5 (7.7*%*)*	*5 (14.7*%*)*	*10 (19.6*%*)*	*6 (35.3*%*)*

Words and figures in italics indicate significant differences among the groups

**Fig. 2:**
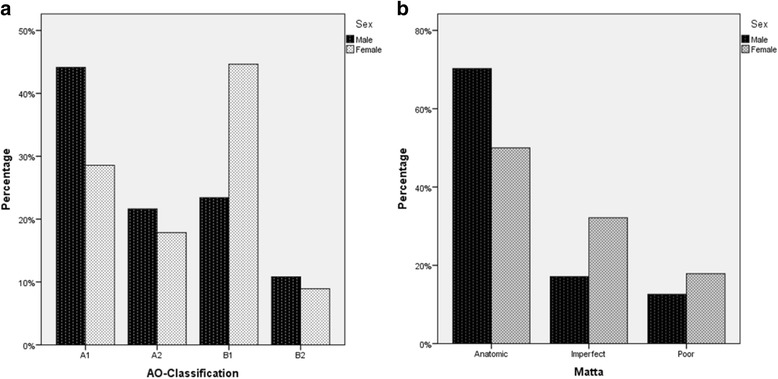
Gender differences in **a** fracture types and **b** reduction quality

Figure 2a, b display significant gender differences in acetabular fracture type (*p* = 0.042; 1-β = 0.803) and reduction quality (*p* = 0.032; 1-β = 0.748). Figure 2a shows that A1 was the most frequent fracture type in males (49 out of 111, 44.1%), whereas females mainly suffered a B1 fracture (25 out of 56, 44.6%). However, no differences in Epstein and Brooker grades, posttraumatic arthritis, as well as postoperative complications were found between males and females (*p* ≥ 0.35).

**Fig. 3:**
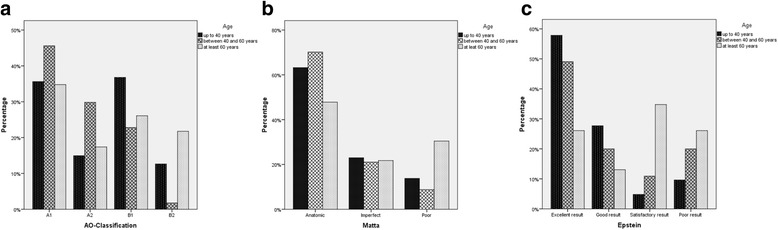
Age-related differences in **a** fracture types, **b** Matta grades, and **c** degenerative changes

For age-specific outcome evaluation, our patient population was subdivided into three groups: patients up to 40 years, patients aged between 40 years and 60 years, and patients with a minimum age of 60 years. The relevant distribution of fracture types is presented in Fig. 3a, which reveals a significant difference (*p* = 0.022; 1-β = 0.970). No significant differences in reduction quality according to Matta could be detected (*p* = 0.150) between the different age groups (see Fig. 3b). In contrast to the Brooker grades (*p* = 0.281), the Epstein grades showed a significantly different distribution in the three age groups (*p* = 0.0005; 1-β = 0.999) which is presented in Fig. 3c. Furthermore, the incidence of infection, revision surgery, avascular necrosis of the femoral head, and posttraumatic arthritis as well as the need of a total hip arthroplasty significantly differed in the three age groups (*p* = 0.005; *p* = 0.001; *p* = 0.022; *p* = 0.0003; *p* = 0.002). Patient numbers and relevant percentages are presented in Table 3.

**Table 3:** Number of complications referring to age

	Patients		
	Up to 40 years	Between 40 and 60 years	At least 60 years
Number of patients	87	57	23
*Infection*	*1 (1.1*%*)*	*3 (5.3*%*)*	*4 (17.4*%*)*
Nerve damage	16 (18.4%)	7 (12.3%)	2 (8.7%)
*Revision surgery*	*1 (1.1*%*)*	*4 (7.0*%*)*	*5 (21.7*%*)*
*THA*	*4 (4.6*%*)*	*6 (10.5*%*)*	*7 (30.4*%*)*
*AVN*	*2 (2.3*%*)*	*3 (5.3*%*)*	*4 (17.4*%*)*
*Postraumatic arthritis*	*9 (10.3*%*)*	*16 (28.1*%*)*	*11 (47.8*%*)*
Hemorrhagic shock	11 (12.6%)	10 (17.5%)	5 (21.7%)

Words and figures in italics indicate significant differences among the groups

Finally, group comparison according to the occurrence of infection (Table 4) identified a higher patient age, a longer interval from accident to surgery, a longer pre-incision time, a longer length of surgery, and a higher intraoperative estimated blood loss in the individuals fighting an infection. However, in individuals suffering a postoperative hemorrhagic shock, higher values were only observed with regard to pre-incision time, length of surgery, and estimated intraoperative blood loss (Table 4).

**Table 4:** Demographic data according to infection and hemorrhagic shock

	Infection			Hemorrhagic shock		
	Yes	No	*p*	Yes	No	*p*
Number	8	157		26	141	
Age (years)	*57.4 ± 14.0*	*41.0 ± 14.6*	*0.002*	43.3 ± 16.1	41.5 ± 14.8	0.570
Interval accident surgery (days)	*12 (6–14)*	*4 (3–7)*	*0.003*	4 (3–7)	4 (3–7)	0.680
Pre-incision time (minutes)	*45 ± 20*	*61 ± 20*	*0.034*	*73 ± 26*	*58 ± 18*	*0.010*
Length of surgery (minutes)	162 ± 60	157 ± 45	0.753	*190 ± 48*	*151 ± 42*	*<0.0001*
Estimated blood loss (mL)	*200 (200–250)*	*300 (200–600)*	*0.023*	*550 (288–800)*	*300 (200–500)*	*0.002*

Data are presented as mean ± standard deviation or median and interquartile range in round brackets

Words and figures in italics indicate significant differences among the groups

## Discussion

The radiographic outcome of patients suffering high-energy acetabular fractures enables conclusions about the functionality and the physical resilience of the restored hip. Reduction accuracy correlates significantly with the clinical outcome [8, 39]. Excellent or good results (assessed with the Harris hip score [40]) were recorded in 89% of the patients with an anatomic reduction, whereas 53% of the patients with a poor reduction had poor clinical results [9]. The quality of reduction is directly related to posttraumatic arthritis; anatomic reduction decreases its incidence [39]. Posttraumatic arthritis is considered as the primary complication following an acetabular fracture and may necessitate a total hip replacement [8]. Undoubtedly, surgical experience is required to achieve the best possible result while avoiding complications. Due to the fact that all surgeries were performed by a single surgeon at a single level I trauma center, a sound foundation could be expected to evaluate different groups without any bias caused by technical skills and individual preferences.

As Fig. 1a shows, the accuracy of reduction was strongly correlated with the fracture type according to the AO/ASIF-classification. Therefore, a comparison with other studies should be treated with caution. Whereas Tannast et al. [5] reported 82% of anatomic reduction for acetabular fractures treated via the Kocher-Langenbeck approach, Alexa et al. [24] achieved anatomic reduction in 59.5% of the fractures. This value is in line with our 63.5%.

Displacement of fracture fragments leads to articular incongruity of the hip joint and therefore to an abnormal pressure distribution on the articular cartilage surface, resulting in a breakdown of the cartilage surface. Figure 1b graphically displays a significantly different distribution of the Epstein grades for the four fracture types. Early radiographic findings in posttraumatic arthritis are minimal narrowing of the joint space and minimal spur formation at the junction of the femoral head-neck. Both abnormalities are evaluated by the Epstein grades just like the femoral head density. Its variation with minimal depression over the weight-bearing part of the femoral head may be an indicator of early avascular necrosis [32]. 21.6% of our patients suffered posttraumatic arthritis and 5.4% AVN. These values are very similar to those presented in the meta-analysis of Giannoudis et al. [3] (19.8 and 5.6%, respectively). Furthermore, the percentages of infections were almost equal (4.8 vs. 4.4% [3]). Finally, 16 (9.6%) of our patients suffered traumatic and 9 (5.4%) of our patients iatrogenic nerve injuries. These values were lower than those (16.4 and 8.0%, respectively) published by Giannoudis et al. [3].

Severe development of heterotopic ossification following acetabular fracture surgery has been correlated with worse functional outcomes and may be associated with increased postoperative pain [41]. Furthermore, race is an important factor; African American patients are significantly more likely to develop severe heterotopic ossification when compared to Caucasian patients [42]. Whereas an incidence of 22.7% was reported by Letournel and Judet [39] for the Kocher-Langenbeck approach, 37.3% of our patients showed signs of heterotopic ossification.

Considering the complications (see Table 2), only posttraumatic arthritis and the occurrence of postoperative hemorrhagic shock differed significantly depending on the fracture type. The reason might be the fact that the radiographic outcome deteriorates with increasing fracture severity, resulting in an inferior quality of reduction and in a longer surgery time, which can lead to arthritis and extensive blood loss, respectively.

The degrees of fracture displacement, fracture comminution, and articular impaction do not only depend on the magnitude and direction of the applied force but also on the strength of the underlying bone. Therefore, we subdivided our patients with a mean age of 41.8 years (males 41.0 years, females 43.3 years) according to gender. The percentage of male patients was 66.5. These values correspond to the relevant values presented in the literature (mean overall age 38.6 years; percentage of males 69.4% [3]). However, gender differences exist in morphologic parameters [43] and in bony geometry. The cup of the acetabulum is oriented more laterally in men than in women. In contrast, females have a greater average acetabular depth and significantly smaller femoral head diameters than males, thereby stabilizing the hip against loading through the femur during a frontal crash. The presence of a larger femoral head diameter in males might result in more eccentric loading of the acetabulum, decreasing the posterior wall’s ability to tolerate crash forces [44]. The different genetic condition might—to some extent—explain the larger number of men suffering an acetabular fracture and maybe also the unequal distribution of fracture types in men and women. Nevertheless, there may be more valid reasons for these findings like fracture etiology, risk-behavior, or body posture. Gender differences in preferred seating and driver position have already been revealed [44]. During a frontal crash, the seating position may affect the contact area between femoral head and acetabulum and therefore the extent of adduction, internal rotation, and flexion at the hip joint which determines the acetabular fracture type [39]. The significantly different quality of reduction (see Fig. 3b) in males and females might result from the significantly different distribution of surgically treated fracture types. Studies have demonstrated that male gender is associated with increased heterotopic ossification formation [45] and extensive blood loss [46]. Based on our experience, we could not confirm these findings.

Finally, we subdivided our patients into three age groups (up to 40 years, between 40 and 60 years, at least 60 years). Figure 3a shows significant age-related differences in fracture types. They might be caused by the change of personal preferences and interests during the course of a person’s life. Probably older patients are also less willing to take risks while practicing sports and leisure time activities. Whereas patient age is reported to be significantly correlated with accuracy of reduction in several studies [9, 47], the Matta grades did not differ significantly between our three age groups (see Fig. 3b). Solely in the Epstein grades, we could provide evidence for age-dependent differences (see Fig. 3c). Nevertheless, complications like infection, revision surgery, need of a total hip arthroplasty, avascular necrosis of the femoral head, and posttraumatic arthritis occur significantly more often in older patients, as Table 3 reveals, indicating that age has to be considered as a parameter that negatively influences the outcome after ORIF of acetabular fractures with high probability.

Limitations of our study include the fact that our mean follow-up period of 10 months was relatively short. Patients did not return for follow-up examinations not only due to changes of residence and work-related reasons, but also maybe because they were satisfied with their surgeries and did not need any further treatment.

## Conclusions

Acetabular fractures are rare injuries in a heterogeneous patient population. We could identify fracture type, gender, and age as prognostic factors for the outcome after ORIF using the Kocher-Langenbeck approach. Due to the fact that many fracture types exist and that they require different treatment regimen with varying prospects of success, presented results always depend on patient selection. Therefore, multicenter trials with large homogenous sample populations are necessary in order to collect sufficient data for general recommendations. Additionally, biomechanical research is inevitable to understand the response and tolerance of the knee-thigh-hip-complex for knee loading conditions.

## Additional file

Additional file 1: Set of data. (SAV 12 kb)

## Abbreviations

AVN: Avascular necrosis of the femoral head
IQR: Interquartile range
ORIF: Open reduction and internal fixation
THA: Total hip arthroplasty
